# Gene transferability and sociality do not correlate with gene connectivity

**DOI:** 10.1098/rspb.2022.1819

**Published:** 2022-11-30

**Authors:** Chunhui Hao, Anna E. Dewar, Stuart A. West, Melanie Ghoul

**Affiliations:** Department of Biology, University of Oxford, Oxford OX1 3SZ, UK

**Keywords:** gene connectivity, horizontal transfer, plasmid mobility, cooperative genes

## Abstract

The connectivity of a gene, defined as the number of interactions a gene's product has with other genes' products, is a key characteristic of a gene. In prokaryotes, the complexity hypothesis predicts that genes which undergo more frequent horizontal transfer will be less connected than genes which are only very rarely transferred. We tested the role of horizontal gene transfer, and other potentially important factors, by examining the connectivity of chromosomal and plasmid genes, across 134 diverse prokaryotic species. We found that (i) genes on plasmids were less connected than genes on chromosomes; (ii) connectivity of plasmid genes was not correlated with plasmid mobility; and (iii) the sociality of genes (cooperative or private) was not correlated with gene connectivity.

## 1. Introduction

Genes interact with one another through the associations between their protein products. Such protein products and their interactions form a protein–protein interaction (PPI) network, where the proteins are the nodes, and the interactions are the edges [1–3]. The connectivity of a gene, usually defined as the number of links a gene's product has to other genes' products, is one of the most elementary characteristics of a gene in its corresponding PPI network [2,4–6]. Gene connectivity varies significantly among different genes. For instance, in an *Escherichia coli* PPI network, the most connected gene has 175 interactions with other genes, while 785 genes have only one interaction [7,8]. In many cases, such variations reflect differences in the essentiality of genes. Genes with higher connectivity are often more essential for the reproductive success of a cell or organism [4,5,9–11].

In prokaryotic microorganisms, gene essentiality may not be the only factor linked to gene connectivity. Prokaryotic genes are often subjected to frequent horizontal gene transfer [12–16], and one therefore might expect that this could also affect their gene connectivity. It has been suggested that highly connected genes should not be carried in parts of the genome that can undergo horizontal gene transfer. This is because if a highly connected gene was transferred to a new host, it would likely be non-functional without the other genes it relies on. This idea, known as the ‘complexity hypothesis’, has been supported by several studies, which found an inverse correlation between the rate of horizontal gene transfer and gene connectivity [17–20]. In these studies, the rate of horizontal gene transfer was estimated by inferring gene gain and loss events from phyletic patterns, and so combined genes that could be gained or lost in several different ways, such as duplications, deletion and inversions [17–20].

**Figure 1.**
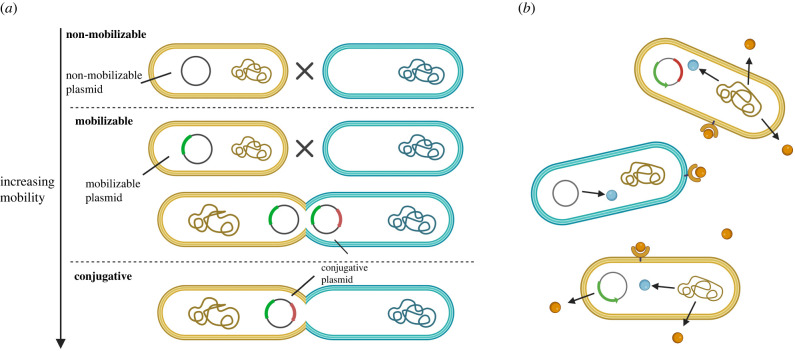
Plasmid mobility and extracellular proteins. (*a*) Plasmid mobility. There are three mobility types of plasmids: non-mobilizable (lowest or no mobility); mobilizable (intermediate mobility) and conjugative (highest mobility). Yellow cells are plasmid donors, while blue cells are plasmid recipients. Each section shows when plasmid transfer can be performed for one of the three plasmid mobility types. Non-mobilizable plasmids cannot be transferred via conjugation. Mobilizable plasmids cannot be transferred alone but can ‘hijack’ the machinery produced by conjugative plasmids. Conjugative plasmids can be transferred independently. (*b*) Extracellular and intracellular proteins. Orange molecules are proteins, which be released outside of cells (extracellular proteins); blue molecules are proteins that only act within cells (intracellular proteins). Yellow cells produce both extracellular proteins and intracellular proteins, while blue cell only produces intracellular proteins, but can receive extracellular proteins produced by other cells. Extracellular proteins provide benefits not only to the cells that produced them, but also to their neighbours (public goods). Created with BioRender.com. (Online version in colour.)

An alternative approach is to compare genes which are maintained or transferred in different ways. We focus on the comparison between genes which are housed on the chromosome, and so likely to have low rates of horizontal gene transfer, versus genes which are housed on plasmids, which represent key vectors of horizontal gene transfer [12,13,21]. Plasmids are self-replicating genetic structures, many of which can move between cells horizontally via a process called conjugation, in addition to vertically via offspring. However, not all plasmids can transfer via conjugation, and so plasmids can be divided into three broad mobility types: non-mobilizable (lowest or no mobility); mobilizable (intermediate mobility) and conjugative (highest mobility) (figure 1*a*) [22,23]. Conjugative plasmids carry all the genes necessary for their transfer [24]. Mobilizable plasmids cannot be transferred alone, but they carry enough genes to ‘hijack’ the machinery of a conjugative plasmid in the same cell [22]. Non-mobilizable plasmids cannot be transferred by conjugation, but only by transduction and transformation like all genes in the genome [22,25].

Another possible factor that may influence gene connectivity is gene function. If certain types of genes are more likely to be transferred horizontally, these types of genes are less likely to have high connectivity, since high connectivity will decrease the likelihood that they are functional when they enter new hosts. For example, it has been suggested that genes which code for extracellular proteins (public goods) are more likely to be transferred horizontally [26,27]. This prediction can arise for two reasons. First, extracellular factors, though not all of them, could provide benefits to other cells, not just those that produced them, and so can represent cooperative helping traits (figure 1*b*). Horizontal gene transfer could favour cooperation, by allowing cooperative genes to reinfect ‘cheats’ that don't produce the extracellular factors (non-cooperators) [28–31]. Second, extracellular factors can allow adaptation to different environments, favouring the ability to gain and/or lose them in different environments. For both scenarios, the complexity hypothesis would predict that genes coding for extracellular proteins may have particularly low connectivity [32].

We carried out an across-species comparative analyses, examining connectivity in 140 genomes from 134 prokaryote species. We asked the following three questions. (i) Are chromosomal genes more connected than plasmids genes? (ii) Do genes on plasmids with higher transfer rates have lower connectivity? (iii) Does gene connectivity vary across genes coding for the production of extracellular versus intracellular factors, and does this vary depending upon whether a gene is on a plasmid or the chromosome?

## 2. Methods

### (a) Network data collection

We extracted PPI networks from the STRING database version 11.0 [33] (https://string-db.org/) and used PPI networks to calculate connectivity. We chose STRING because it covers a large number of organisms (5090), allowing for across-species comparative analysis. In addition, STRING is a comprehensive PPI network database: unlike other databases based on either experimental [34–37], or computational prediction interactions [38], STRING integrates both of these and includes direct (physical) and indirect (functional) associations. This allowed us to include as many prokaryotic species as possible in our analyses.

The evidence for each interaction in the STRING database is categorized into one of seven independent ‘channels’: neighbourhood, fusion, co-occurrence, co-expression, text-mining, experiments and databases. Proteins can functionally interact without touching, such as when a transcription factor helps to regulate the expression and production of another protein, or when two enzymes exchange a specific substrate via diffusion [33]. Such indirect interactions could be inferred, for example, from co-expression or co-occurrence patterns between genes. For each pair of interactions, a separate score is given per channel. A combined confidence score ranging from 0 to 1000 is given by combining and adjusting the scores from the different channels [39]. The larger the threshold, the higher the confidence score, but it also means fewer proteins, interactions and species. In our main analysis, we specified a threshold of 400 for the combined scores of the interactions, meaning any interaction below this threshold would not be considered. 400 is a medium confidence threshold according to the STRING database. We chose this threshold for our main analysis to gain a balance between confidence and sample size. To check the reproducibility of our results, we also repeated our analysis by setting three other thresholds: 150 (low confidence), 700 (high confidence) and 900 (highest confidence). The results at different thresholds are presented in the electronic supplementary material, tables. To match with other databases, we retrieved all the available PPI networks by using the STRINGdb package (version 2.4.0) in R [33] (see 'Database Matching and Genome Collection' below).

### (b) Categorization of genes and annotations of replicons

To select genes that were putatively ‘cooperative’, we followed the methods of previous studies which have considered genes coding for extracellular proteins as a proxy for ‘cooperative’ genes [40–42]. This is because extracellular proteins often act as public goods, whose benefits are shared with neighbouring cells [27]. Although not all cooperative genes produce extracellular proteins and not all extracellular proteins are cooperative, any strong effect of sociality is likely to be captured by using this proxy [32]. We determined protein subcellular localization for each protein included in our analysis with PSORTdb 4.0 (https://db.psort.org/) [43]. PSORTdb was selected for its reliability and validity in systematically deducing both bacterial and archaeal protein subcellular localization.

PSORTdb gives a final prediction of the subcellular location for each protein. For Gram-positive bacteria, the program allocates proteins to one of four locations within the cell: cytoplasmic, cytoplasmic membrane, extracellular or cell wall. Many of the most well-studied Archaea contain the same basic components as classic Gram-positive bacteria [43]. For Gram-negative bacteria, proteins are assigned to one of five locations, where the cell wall has been replaced by the outer membrane or periplasmic. We excluded any proteins classified as ‘unknown’ by PSORTdb from our analysis, which accounted for 23.9% of all proteins we analysed.

The PSORTdb outputs we used also included whether each gene was carried on a plasmid or a chromosome. We initially collected precomputed PSORTdb results for all available genomes, including 73 136 replicons belonging to 8416 bacterial and archaeal strains, and kept all genomes that were also in the STRINGdb with a PPI network. All the PSORTdb results were retrieved and compiled using GNU Wget and R.

### (c) Database matching and genome collection

To compare the connectivity (see below) of genes encoding extracellular and intracellular proteins, we curated a list of bacterial and archaeal strains which were in both the PSORTdb and STRING databases. We used NCBI Entrez Direct Command Line Tools (https://www.ncbi.nlm.nih.gov/books/NBK179288/) to transfer NCBI taxonomy IDs used by STRING, to RefSeq genome/replicon accessions used by PSORTb. By doing so, we were able to extract the PSORTdb results of all genomes which also had a PPI network(s). To allow us to compare chromosome and plasmid genes, we only considered genomes with PSORTdb results that included at least one plasmid sequence. Specifically, for our purpose of comparing the connectivity of genes coding for extracellular and intracellular proteins that are on plasmids, we omitted genomes with no extracellular protein-coding genes on their plasmids. A total of 1570 strains were retrieved originally, of which 462 strains had gene connectivity data on chromosomes and plasmids, and 167 strains had connectivity data on genes encoding extracellular proteins on chromosomes and plasmids. To make sure all species in our dataset were unique, we only included species with a complete Latin binomial name. This gave us a list of 134 species (140 genomes), which included 5 archaeal species (5 genomes) and 129 bacterial species (135 genomes), with 358 plasmid genomes in total (electronic supplementary material, tables S1 and S2).

For each gene in our dataset, we mapped the gene name to the STRING database identifier ‘STRING_id’ using the ‘map’ function of the R package STRINGdb version 2.6.1 [33]. This unique ‘STRING_id’ was used to calculate the connectivity for every individual gene. Genes that could not be mapped with ‘STRING_id’ were not included in our dataset. Details on the number of genes per genome (strain) per species included in this analysis could be found in the electronic supplementary material, table S8.

### (d) Connectivity

The complexity hypothesis suggests that highly connected genes are less likely to undergo horizontal transfer, because if a highly connected gene was transferred to a new host, it would probably be non-functional without the other genes it relies on. To examine the connectivity of genes, we used the term ‘gene connectivity’ to mean the same as the ‘protein connectivity’ of its protein product in a PPI network. We followed previous studies by defining protein connectivity as the number of PPIs in which the protein is embedded in the PPI network [17,44]. We used this definition because one fundamental assumption of the complexity hypothesis is that the more interactions a gene has with other genes, the more complex it is, and therefore the less likely the gene will be functional when transferred to a new host [20].

The connectivity of each gene was different in networks with different thresholds. Because when a specific network threshold was used, any PPIs below this threshold were not considered, resulting in some genes being omitted if their protein products did not interact with any other proteins above this threshold. As the network threshold increased, the number of genes and interactions included in the analyses decreased. The connectivity of each gene was also reduced. Details on the number of genes per genome per species included in our analyses of networks with different thresholds, and the average connectivity of all genes per genome per species could be found in the electronic supplementary material, table S8.

Network size (the total number of proteins in a PPI network) could influence gene connectivity [45]. Genes with the same connectivity have different impacts in networks of different sizes. To control for the possible influence of network size in our analyses, we also examined whether network size was correlated with the connectivity of genes in our dataset. We performed all calculations of connectivity using the R package ‘igragh’ [46].

### (e) Predicting plasmid mobility and host range

To predict the mobility of every plasmid in our dataset, we used the MOB-typer tool of the software MOB-suite [47]. This tool is designed to provide *in silico* predictions of the origin of transfer (oriT), relaxase type and mate-pair formation (MPF) type for each plasmid based on its sequence. Afterwards, each plasmid is assigned to one of three mobility types: (i) conjugative, where the plasmid contains the complete set of genes and DNA features needed for transfer; (ii) mobilizable, where the plasmid encodes either a relaxase or an oriT but is missing the MPF marker and (iii) non-mobilizable, where plasmid is missing a relaxase and an oriT [47]. We classified the mobility of 358 plasmids for subsequent analysis (electronic supplementary material, table S2). MOB-suite also provided information on plasmid's host range, which is a measure of the breadth of the different bacterial hosts a plasmid is carried in. Specifically, it is defined as the highest taxonomic rank of the genomes in which a plasmid is found. For example, a plasmid found only in genomes of *Yersinia* sp. would have a host range of ‘genus’, while a plasmid found in a number of Gammaproteobacteria species would have a host range of ‘class’. In general, the higher the taxonomic rank of genomes carrying the plasmid, the larger the plasmid's host range. Each plasmid was assigned one of six plasmid's host ranges: genus, family, order, class, phylum and multi-phyla (electronic supplementary material, table S2).

### (f) Statistics

We carried out all statistical analyses and graph plotting in R (v. 4.0.2). For all comparisons between groups that included all our species, we used the R package MCMCglmm [48]. MCMCglmm fits generalized linear mixed-effects models (GLMMs) using a Markov chain Monte Carlo approach under a Bayesian statistical framework [48]. Species share traits descended from their common ancestor, and so cannot be considered as independent data points. We thus used MCMCglmm to control for this, with a phylogeny as a random effect in our models (see 'Phylogeny' below) [49]. For each analysis, we used 1 100 000 model iterations with a starting burn-out phase of 100 000, sampling every 1000 iterations. We then checked the reliability of all output models by looking at model convergence. After the model diagnoses, we reported the posterior mean, 95% credible intervals (functionally similar to 95% confidence intervals), and the pMCMC value (used here as ‘*p*-value’) for each model. We also provided the *R*^2^ for our main analyses using methods described in [50,51]. DIC is a hierarchical modelling generalization of the Akaike information criterion, which balances model fit and model complexity simultaneously. Like other information criteria, smaller values of DIC are preferred [48].

### (g) Phylogeny

To control for phylogenetic relationships between our species, we used a phylogenetic tree including all 134 species in our dataset (electronic supplementary material, figure S6). We put together this phylogeny using the methods of a recent study [32]. The tree was based on a recently published maximum-likelihood tree of life using 16 ribosomal protein sequences data [52]. This tree typically has only one representative species of each genus. We first extracted all branches that matched species in our dataset by using the R package ‘ape’ [53]. In cases where the representative species of a genus was not the same as our species from the same genus, we replaced the branch tip with our species, since all species from the same genus are equally related to species of sister genera. In cases where there were two species per genus in our dataset, we used the R package ‘phylotools’ to directly add the second species as an additional branch into their genera [54]. Where there were more than two species within a genus in our dataset, we consulted phylogenies from the literature to add any within-genus clustering of species' branches.

## 3. Results

### (a) Chromosomal genes are more connected than plasmid genes

**Figure 2.**
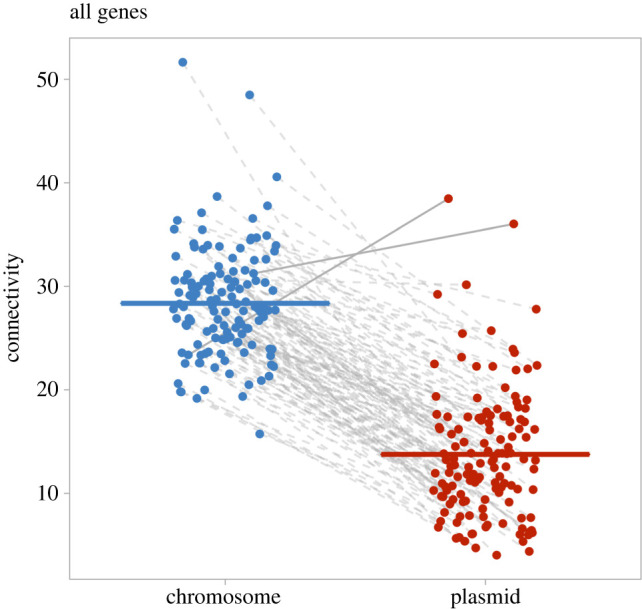
The relative connectivity between chromosomal genes and plasmid genes. Each dot represents the average connectivity of all genes in either the chromosome or plasmid(s) of one species. Chromosome and plasmid values of the same species are linked by a line. A solid line means the average connectivity of chromosomal genes is lower than that of plasmid genes, while a dashed line means the average connectivity of chromosomal genes is higher than that of plasmid genes. The two horizontal lines represent the mean for each group. For almost all species (132/134), chromosomal genes have a higher level of connectivity than plasmid genes. (Online version in colour.)

We first compared the gene connectivity between chromosomal genes and plasmids genes. For our main analysis using networks with a medium threshold of confidence, we found that genes located on chromosomes had significantly higher levels of connectivity compared to genes on plasmids (figure 2). Specifically, across species, the difference in connectivity between chromosomal genes and plasmid genes was significantly different from zero (MCMCglmm [48]; posterior mean = 15.127, 95% CI = 12.061 to 18.169, pMCMC < 0.001, *n* = 134 species, *R*^2^ of phylogeny = 0.201; figure 2; electronic supplementary material, table S3).

Our result was robust to alternative analyses. First, we found the same pattern when we instead analysed the ratio of connectivity between chromosomal genes and plasmid genes instead of the difference. Chromosomal genes on average interacted with 2.6 times more genes than plasmid genes (MCMCglmm; posterior mean = 2.58, 95% CI = 2.002 to 3.142, pMCMC < 0.001, *n* = 134 species, *R*^2^ of phylogeny = 0.36; electronic supplementary material, table S3). Second, when we looked at the individual species level, chromosomal genes had higher connectivity than plasmid genes in 98.5% of species (132/134); only *Beijerinckia indica* and *Ralstonia pickettii* had plasmid genes with higher connectivity (electronic supplementary material, figure S1 and table S4). Third, we obtained the same qualitative result when we used networks with different confidence thresholds of PPIs (electronic supplementary material, table S3). Increasing the threshold reduced the posterior mean of the differences in chromosome and plasmid connectivity and also reduced the number of species included (electronic supplementary material, table S3).

### (b) Plasmid mobility does not affect the relative gene connectivity between chromosomes and plasmids

**Figure 3.**
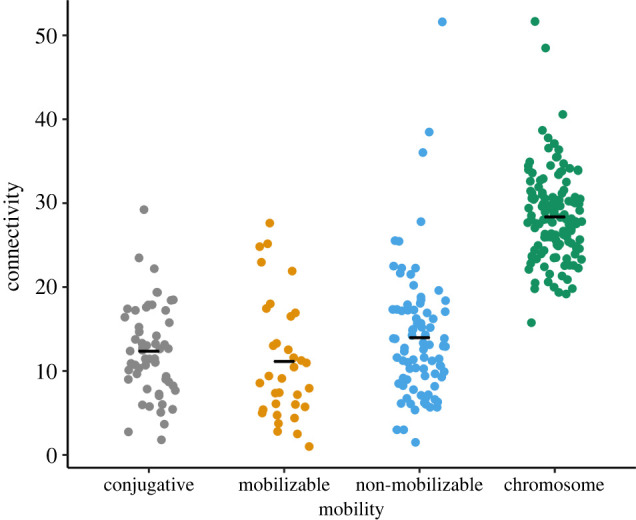
Connectivity of genes on plasmids with different mobilities. We classified plasmids into three mobility types: conjugative (highest mobility); mobilizable (intermediate mobility) and non-mobilizable (lowest mobility). Each dot represents the mean connectivity of all genes on certain replicons for one species. The black horizontal line represents the mean for each group. Plasmid mobility does not influence relative gene connectivity between chromosomes and plasmids. (Online version in colour.)

We then examined whether the mobility of plasmids was correlated with connectivity. We assigned each plasmid in our dataset with one of three mobility types using MOB-suite [47]: non-mobilizable (lowest mobility); mobilizable (intermediate mobility) and conjugative (highest mobility). We found genes on plasmids with different mobilities did not differ from each other in gene connectivity when compared to genes on chromosomes. Specifically, we first compared the gene connectivity between chromosomes and plasmids across these three mobility types, and found that genes on all three types of plasmids had significantly lower connectivity than genes on chromosomes (MCMCglmm; conjugative plasmids compared to chromosomes: posterior mean = −17.03, 95% CI = −18.60 to −15.24, pMCMC < 0.001; mobilizable plasmids compared to chromosomes: posterior mean = −16.87, 95% CI = −19.03 to −14.81, pMCMC < 0.001; non-mobilizable plasmids compared to chromosomes: posterior mean = −14.15, 95% CI = −15.61 to −12.69, pMCMC < 0.001; conjugative plasmids compared to mobilizable plasmids: posterior mean = 0.20, 95% CI = −2.23 to 2.46, pMCMC = 0.87; mobilizable plasmids compared to non-mobilizable plasmids: posterior mean = 2.75, 95% CI = 0.59 to 5.30, pMCMC = 0.028; *n* = 134 species; *R*^2^ of fixed effect = 0.535; DIC = 1943.26; figure 3; electronic supplementary material, table S3). Second, we generated a minimum adequate model by combining all genes on plasmids with different mobilities, and compared their gene connectivity with genes on chromosomes. We found that plasmids genes were significantly less connected than chromosomal genes (MCMCglmm; posterior mean = −14.67, 95% CI = −15.85 to −13.67, pMCMC < 0.001; *R*^2^ of fixed effect = 0.572; DIC = 1698.59; figure 2; electronic supplementary material, table S3). Finally, by comparing *R*^2^ of fixed effect and DIC between the two models, we preferred the second minimum adequate model with a higher *R*^2^ of fixed effect and lower DIC. These results suggested that plasmid mobility did not influence relative gene connectivity between chromosomes and plasmids. This result was robust to alternative analysis when we looked at the patterns in networks with different confidence thresholds of PPIs (electronic supplementary material, table S3).

Additionally, we also carried out analysis to examine whether the host range of plasmids was correlated with connectivity. We assigned each plasmid in our dataset with one of the six host ranges using MOB-suite [47]: genus, family, order, class, phylum and multi-phyla. We then compared the gene connectivity of genes on plasmids with different host ranges and genes on chromosomes. We found that genes on plasmids with different host ranges did not differ from each other in gene connectivity when compared to genes on chromosomes (MCMCglmm; multi-phyla compared to phylum: posterior mean = 2.30, 95% CI = −7.39 to 12.66, pMCMC = 0.66; multi-phyla compared to class: posterior mean = −0.90, 95% CI = −8.70 to 8.05, pMCMC = 0.83; multi-phyla compared to order: posterior mean = 8.04, 95% CI = −0.12 to 16.21, pMCMC = 0.06; multi-phyla compared to family: posterior mean = 2.37, 95% CI = −6.71 to 10.72, pMCMC = 0.57; multi-phyla compared to genus: posterior mean = 6.22, 95% CI = −1.63 to 14.36, pMCMC = 0.13; multi-phyla compared to chromosomes: posterior mean = 21.69, 95% CI = 13.53 to 29.59, pMCMC < 0.001; electronic supplementary material, figure S7 and table S3). This result was robust to alternative analysis using networks with different thresholds (electronic supplementary material, table S3).

### (c) Genes connectivity did not consistently differ between genes encoding extracellular or intracellular proteins

**Figure 4.**
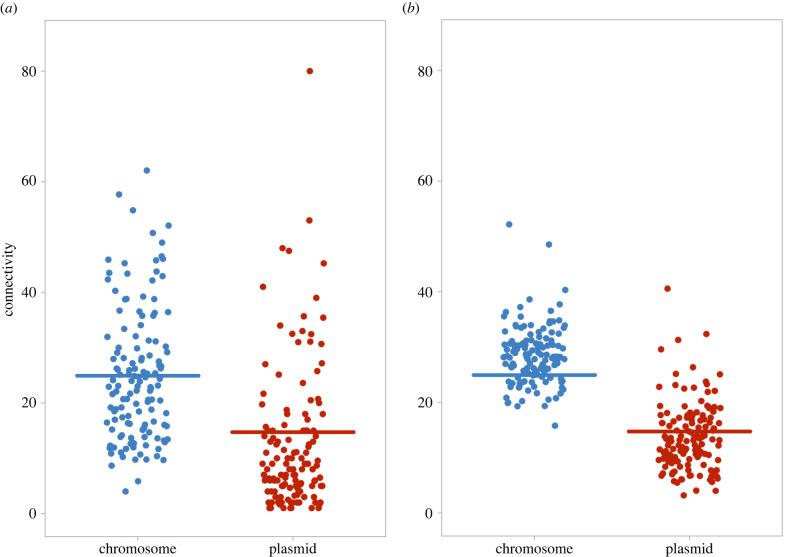
Comparison of chromosomal and plasmid connectivity for genes with different sociality (*a*) chromosome versus plasmid comparisons for genes encoding extracellular proteins (cooperative); (*b*) chromosome versus plasmid comparisons for genes encoding intracellular proteins (private). Each dot represents the mean connectivity of all genes with certain types of protein products for one species. The horizontal line represents the mean for each group. Two outlying species have been removed from figure 4*b*, where genes on plasmids have much higher levels of connectivity (greater than 85). The complete version of figure 4*b*, with these two outlying data points, is in the electronic supplementary material, figure S4. Chromosomal genes were more connected than plasmid genes, for both genes encoding extracellular proteins and genes encoding intracellular proteins. (Online version in colour.)

We then tested if genes coding for extracellular (cooperative) and intracellular (private) proteins were different from each other in terms of their relative gene connectivity between chromosomes and plasmids. We found genes coding for extracellular proteins (cooperative) were significantly more connected on chromosomes than on plasmids (MCMCglmm; posterior mean = 9.325, 95% CI = 0.108 to 17.203, pMCMC = 0.032; *n* = 134 species; figure 4*a*; electronic supplementary material, figure S4 and table S3). At the individual species level, 81.3% (109/134) of species showed this pattern, with genes coding for extracellular proteins having higher connectivity on chromosomes than plasmids (electronic supplementary material, figure S2 and table S5). Genes coding for intracellular proteins (private) were also significantly more connected on chromosomes than on plasmids (MCMCglmm; posterior mean = 15.325, 95% CI = 12.190 to 18.393, pMCMC < 0.001; *n* = 134 species; figure 4*b*; electronic supplementary material, figure S4 and table S3). At the individual species level, 97.8% (131/134) of species showed this pattern in that direction (electronic supplementary material, figure S3 and table S6).

We then examined sociality (extracellular or intracellular) and location (chromosome or plasmid) simultaneously. We found that the difference in gene connectivity between chromosomal genes and plasmids genes was smaller for genes coding for extracellular proteins compared to intracellular proteins (MCMCglmm; Interaction term: posterior mean = −4.410, 95% CI = −8.567 to −0.331, pMCMC = 0.042; *n* = 134 species; *R*^2^ of interaction term = 0.021, electronic supplementary material, table S3). The small *R*^2^ of the interaction term suggested that the effect size of the interaction between sociality and location is small. In addition, the significance of this result was not robust to different network thresholds, with the interactions between sociality and location being significant at the lower threshold (150), but not at higher thresholds (700 and 900). Although the interaction between sociality and location was significant in networks with a threshold of 150, the effect size of the interaction remained small (MCMCglmm; Interaction term: posterior mean = −23.096, 95% CI = −43.832 to −2.354, pMCMC = 0.042; *n* = 134 species; *R*^2^ of interaction term = 0.004, electronic supplementary material, table S3). Overall, these results suggest that there is either a small or no interaction between the influence of sociality and location on gene connectivity (electronic supplementary material, table S3). If we ignored the potential interactions between sociality and location when in associate with gene connectivity, and directly compared the connectivity between genes coding for extracellular and intracellular proteins, we found no significant differences between them (MCMCglmm; posterior mean = −6.06, 95% CI = −12.72 to −0.68, pMCMC = 0.052; *n* = 134 species; figure 4).

We also found a suggestive result that the variance of gene connectivity was greater for extracellular proteins, relative to intracellular proteins. This result is only suggestive because we were only able to examine with a standard *F*-test (*F*-test; chromosomes: *F*_133,133_ = 0.194, *p*-value < 2.2 × 10^−16^; plasmids: *F*_133,133_ = 0.084, *p*-value < 2.2 × 10^−16^), rather than a MCMCglmm analysis that controls for phylogeny. Species often share characteristics inherited through common descent, rather than through independent evolution and so cannot be considered independent data points [55]. Therefore, given that we cannot control for phylogeny, this result should only be seen as suggestive.

### (d) Network size did not affect our results

Network size (the total number of proteins in a PPI network) could affect gene connectivity. In the extreme, gene connectivity could be constrained by small network sizes. There is evidence that genes with the same connectivity have different impacts in networks of different sizes [45]. To examine whether network size influenced the connectivity of genes in our dataset, we tested whether the two were correlated. We found no significant correlation between network size and gene connectivity (MCMCglmm; posterior mean = 0.000212, 95% CI = −0.000480 to 0.000916, pMCMC = 0.578, *n* = 134 species; electronic supplementary material, figure S5 and table S7). We also found no significant correlation between network size and either the difference or the ratio of connectivity between chromosomes and plasmids (electronic supplementary material, table S7). The same results were also applied to genes coding for extracellular proteins (electronic supplementary material, table S7). These results suggest that network size did not affect our finding that connectivity of genes is higher for chromosomes than plasmids.

### (e) Gene connectivity was higher in core versus accessory genes

We also compared the gene connectivity between core genes, that are found in all (100%) genomes of a species, and accessory genes, which are only found in some genomes. The pangenome information was retrieved from the PanX database (https://pangenome.org/) [56]. We were only able to analyse seven species (nine strains) in our dataset where there was also data available on core and accessory genes in the PanX database (electronic supplementary material, table S9). We found that when on chromosomes, core genes had significantly higher levels of connectivity compared to accessory genes (MCMCglmm; posterior mean = 12.63, 95% CI = 7.22 to 17.68, pMCMC < 0.001, *n* = 7 species; electronic supplementary material, figure S8 and table S3). This result was also robust to networks with other thresholds (electronic supplementary material, table S3).

## 4. Discussion

We found that: (i) as predicted by the complexity hypothesis, plasmid genes had consistently lower connectivity compared to chromosome genes (figure 2); (ii) contrary to the prediction of the complexity hypothesis, there was no correlation between plasmid mobility and gene connectivity (figure 3); and (iii) genes encoding extracellular proteins and genes encoding intracellular proteins did not differ in relative gene connectivity between chromosomes and plasmids (figure 4).

Our finding that genes on plasmids had lower levels of connectivity than genes on the chromosome is consistent with previous studies looking at horizontally transferred genes [17–19] (figure 2). The explanation of this pattern from the perspective of the complexity hypothesis is that if genes on plasmids are likely to undergo frequent horizontal gene transfer, they will be less likely to be malfunctioning in a new host if they have lower connectivity [17,20]. However, alternative explanations are also possible, because plasmids are not merely gene delivery platforms and not all plasmids are transferable [57]. Around 53% of plasmids lack relaxases (i.e. non-mobilizable plasmids), thus cannot be mobilized by conjugation, but only by transformation or transduction, similar to chromosomal genes [24]. Even for mobilizable plasmids that are capable of using the genetic cassette of other conjugative plasmids, the rate of conjugation of these plasmids is still likely to be lower than that of conjugative plasmids [58–60]. Therefore, if plasmid genes were less connected because they undergo more frequent horizontal gene transfer, as predicted by the complexity hypothesis, we would expect plasmid gene connectivity to decrease with increasing plasmid mobility.

Our next finding, however, suggested that in contrast with the complexity hypothesis, the mobility of plasmids was not correlated with gene connectivity (figure 3). This suggests that other evolutionary forces beyond the horizontal transfer of plasmids may contribute to the pattern we observed. This is also in line with a recent review which highlighted characteristics of plasmids, in addition to their potential for horizontal transfer, that could drive specific evolutionary dynamics of plasmid-encoded genes, and have been largely overlooked until recently [57].

Instead of horizontal gene transfer, another possible factor that could explain the difference between plasmid and chromosome gene connectivity is the inheritance stability of genes on plasmids. Plasmids are suggested to be less stable than chromosomes, because (i) plasmids usually confer a cost to the host, causing a competitive disadvantage that may select for plasmids to be lost in the absence of a benefit to the cell, and (ii) plasmids can be lost during cell division if they are incompletely segregated between daughter cells [21,61–65]. Therefore, genes on plasmids may not be as stable as genes on chromosomes.

A theoretical study suggested that essential genes, which are usually highly connected, were more likely to be found on chromosomes rather than on plasmids because the inheritance of chromosomes is more stable than that for plasmids [66]. A recent empirical study provided further evidence that plasmid inheritance instability is responsible for essential genes not being carried on plasmids, by observing that inserting an essential chromosomal gene into a plasmid makes the plasmid more likely to be lost in *E. coli* [67]. A variety of mechanisms have been suggested to help stabilize plasmid persistence, such as host-plasmid co-adaptation, compensatory evolution and high plasmid transfer rates [65,68–71]. Theory predicts that even low rates of plasmid loss can make essential genes more likely to be on chromosomes than plasmids. Specifically, the only case where essential genes can be found on plasmids is when essential genes are more likely to be lost when on chromosomes compared to on plasmids [66]. However, this is very unlikely to be the case in the real world. Consequently, given our results that plasmid mobility has a limited effect on plasmid gene connectivity, the instability of plasmid inheritance may instead be a more likely explanation for why highly connected genes are frequently absent from plasmids.

Examining chromosomal genes, we found that core genes, present in every genome, had a higher connectivity than accessory genes, found in only a subset of genomes (electronic supplementary material, figure S8 and table S3). This result was predicted because core genes are likely to encode more essential functions than accessory genes, and thus tend to have higher connectivity [4,5]. An alternate explanation, that cannot be separated, is that horizontal gene transfer is more common in the accessory genome. Horizontal gene transfer is just one process for shaping the accessory genome, which accounts for 15.5% of accessory genomes, alongside with other processes such as gene deletion [72,73]. Although these rates of horizontal gene transfer are much lower than with plasmids, and we cannot separate out different rates of horizontal gene transfer as we can with plasmids (figure 1).

Chromosomal genes can undergo horizontal gene transfer via mechanisms such as integrative conjugative elements (ICEs) and prophages [74,75]. However, there are three reasons that this is unlikely to have had a major influence on the broader patterns we examined comparing plasmids and chromosomes. First, most chromosomal genes can be regarded as immobile. A study of 80 bacteria found that horizontally transferred genes were concentrated in only a small fraction of chromosomal regions (approx. 1%) [72]. Second, plasmids have evolved to have fitness interests distinct from chromosomes, therefore, chromosomal and plasmid genes differ not only in their rate of horizontal gene transfer, but also in how selection operates to shape their traits [76]. Regarding gene connectivity, our findings demonstrated that selection was not necessarily associated with the level of horizontal gene transfer across plasmids. Third, rates of horizontal transfer can be much higher for plasmids compared to chromosomes [77,78]. What matters is not just whether horizontal transfer occurs, but how frequently over evolutionary time. Nonetheless, a useful future direction would be to extend analyses to other routes of horizontal transfer, such as ICEs.

We also compared the connectivity of genes encoding cooperative (extracellular) to genes encoding private (intracellular) factors. We examined this factor because genes coding for cooperative factors have been hypothesized to undergo more frequent horizontal transfer and may have particularly low connectivity when on plasmids to allow for easier transfer [29,30]. However, we found no difference in the connectivity of cooperative and private genes, and we also found that the relative difference between plasmids and chromosome genes was the same for cooperative genes compared to private genes (figure 4). This lack of difference in connectivity between genes coding for extracellular and intracellular proteins could be due to two reasons. (i) Genes coding for extracellular proteins are not more likely to be transferred horizontally. A recent study found that genes coding for extracellular proteins were not overrepresented on plasmids compared to chromosomes, or on more mobile plasmids compared to less mobile plasmids [32]. (ii) Other factors could affect gene connectivity of plasmid genes in addition to any effect due to horizontal gene transfer, such as gene essentiality or plasmid instability, which would act similarly on all genes, not just those coding for extracellular proteins. Either way, these results suggested that the factors determining which genes are carried on plasmids, particularly which genes might bear the potential costs of plasmid instability and loss, are not affected by whether the gene codes for a cooperative public good.

To conclude, our results provide mixed support for the complexity hypothesis, suggesting that there are other factors at play. While genes on plasmids have lower connectivity than genes on chromosomes, their connectivity did not correlate with the rate at which different plasmids are likely to transfer horizontally. This suggests that other factors, particularly the stability of gene inheritance, might be more important than gene mobility in explaining the variation in gene connectivity across prokaryotic genomes. Key tasks for the future include (i) examining gene connectivity on other mobile genetic elements (such as phages and ICEs [79]), (ii) directly examining the role of stability and (iii) using different or additional methods for identifying cooperative traits.

## Data Availability

The data used to generate all results and figures are provided in the electronic supplementary material [80].
